# Efficacy of amoxicillin and amoxicillin/clavulanic acid in the prevention of infection and dry socket after third molar extraction. A systematic review and meta-analysis

**DOI:** 10.4317/medoral.21139

**Published:** 2016-03-06

**Authors:** María-Iciar Arteagoitia, Luis Barbier, Joseba Santamaría, Gorka Santamaría, Eva Ramos

**Affiliations:** 1 MD, DDS, PhD, Associate Professor, Stomatology I Department, University of the Basque Country (UPV/EHU), BioCruces Health Research Institute, Spain; Consolidated research group (UPV/EHU IT821-13); 2 MD PhD, Chair Professor, Maxillofacial Surgery Department, BioCruces Health Research Institute, Cruces University Hospital, University of the Basque Country (UPV/EHU), Spain; Consolidated research group (UPV/EHU IT821-13); 3 MD, DDS, PhD, Professor and Chair, Maxillofacial Surgery Department, Bio Cruces Health Research Institute, Cruces University Hospital, University of the Basque Country (UPV/EHU), Bizkaia, Spain; Consolidated research group (UPV/EHU IT821-13); 4 DDS, PhD, Associate Professor, Stomatology I Department, University of the Basque Country (UPV/EHU), BioCruces Health Research Institute, Spain; Consolidated research group (UPV/EHU IT821-13); 5 PhD, Degree in Farmacy, BioCruces Health Research Institute, Cruces University Hospital. Spain

## Abstract

**Background:**

Prophylactic use of amoxicillin and amoxicillin/clavulanic acid, although controversial, is common in routine clinical practice in third molar surgery.

**Material and Methods:**

Our objective was to assess the efficacy of prophylactic amoxicillin with or without clavulanic acid in reducing the incidence of dry socket and/or infection after third molar extraction. We conducted a systematic review and meta-analysis consulting electronic databases and references in retrieved articles. We included double-blind placebo-controlled randomized clinical trials published up to June 2015 investigating the efficacy of amoxicillin with or without clavulanic acid on the incidence of the aforementioned conditions after third molar extraction. Relative risks (RRs) were estimated with a generic inverse-variance approach and a random effect model using Stata/IC 13 and Review Manager Version 5.2. Stratified analysis was performed by antibiotic type.

**Results:**

We included 10 papers in the qualitative review and in the quantitative synthesis (1997 extractions: 1072 in experimental groups and 925 in controls, with 27 and 74 events of dry socket and/or infection, respectively). The overall RR was 0.350 (*p*< 0.001; 95% CI 0.214 to 0.574). We found no evidence of heterogeneity (I2=0%, *p*=0.470). The number needed to treat was 18 (95% CI 13 to 29). Five studies reported adverse reactions (RR=1.188, 95% CI 0.658 to 2.146, *p* =0.567). The RRs were 0.563 for amoxicillin (95% CI 0.295 to 1.08, *p*=0.082) and 0.215 for amoxicillin/clavulanic acid (95% CI 0.117 to 0.395, *p*<0.001).

**Conclusions:**

Prophylactic use of amoxicillin does not significantly reduce the risk of infection and/or dry socket after third molar extraction. With amoxicillin/clavulanic acid, the risk decreases significantly. Nevertheless, considering the number needed to treat, low prevalence of infection, potential adverse reactions to antibiotics and lack of serious complications in placebo groups, the routine prescription of amoxicillin with or without clavulanic acid is not justified.

**Key words:**Meta-analysis, amoxicillin, infection, removal, dry socket, third molar.

## Introduction

Third molar extraction is a common procedure in oral surgery. There still is controversy over the need to routinely use systemic antibiotics for the prevention of infectious and inflammatory complications associated with this type of surgery (1,2).

In a survey in 2014 (3), we found that 83% of dentists in our region (Bizkaia) would administer antibiotics prophylactically for surgery of fully impacted third molars fully covered by bone in healthy patients, the drugs most commonly prescribed being amoxicillin (58.3%) and amoxicillin/clavulanic acid (34.5%). Most reviews and meta-analyses on this topic question the routine use of antibiotics in healthy patients, given that these drugs may cause adverse reactions and that their inappropriate use leads to the development of resistant bacteria (4-9). In order to assess the scientific evidence on the widespread clinical practice among dentists of administering amoxicillin with or without clavulanic acid before or during surgery, we have designed a meta-analysis including all available high quality clinical trials on the prophylactic use of amoxicillin with or without clavulanic acid.

The objectives of this study were; 1: to assess the efficacy of the use of amoxicillin with or without clavulanic acid to prevent infection and/or dry socket, compared to a control group given placebo, in third molar surgery patients; and 2: to carry out stratified analysis of the efficacy of amoxicillin and amoxicillin/clavulanic acid. We designed a meta-analysis testing the null hypothesis that the use of amoxicillin with or without clavulanic acid is not effective.

## Material and Methods

This study is reported in accordance with the Preferred Reporting Items for Systematic Reviews and Meta-Analyses (PRISMA) statement and the Institute of Medicines’ guidelines. The literature search was based on questions structured in the Patient, Intervention, Comparison, and Outcome (PICO) format.

- Eligibility criteria: We selected studies including patients of any age and sex who underwent extraction of third molars with any degree of impaction. Regarding type of intervention, we included trials that analysed the efficacy of amoxicillin with or without clavulanic acid at any dose or regimen, and regarding comparisons, we exclusively included randomized double-blind placebo-controlled (RDBPC) clinical trials, not excluding those with split-mouth designs. With respect to outcome, we excluded studies that did not investigate the incidence of dry socket, infection, and both conditions concurrently, but did not apply restrictive criteria for the definition of infection or dry socket. Results of interest: The search was not restricted by language. The last search date was 1 June 2015.

- Sources of information. The electronic databases consulted were: Medline/PubMed, Scopus, ScienceDirect, Web of Science, Evidence-Based Dentistry, ClinicalTrials.gov, the EU Clinical Trials Register, the Cochrane Central Register of Controlled Trials, the Spanish General University Board database of doctoral theses in Spain (TESEO) and Spanish National Research Council (CSIC) bibliographic databases.

- Search strategy: The search terms selected are descriptors of each of the PICO components: extraction, removal; third molar; antibiotic, amoxicillin, clavula*; infection; and dry socket. The filters used were: humans, clinical trials, meta-analysis, randomised, and controlled trials. The electronic search in the Medline/PubMed database was carried out using MeSH strings and search algorithms connected with Boolean operators as key words for titles and abstracts. Specifically, we used the following search strategy: (randomized controlled trials OR controlled clinical trial OR randomized controlled trials OR random allocation OR double-blind method OR clinical trial OR clinical trials OR) (“clinical trial”) OR (doubl* OR trebl* OR tripl*) AND (mask* OR blind*) OR (“Latin square” ) OR placebos OR placebo* OR random* OR research design OR comparative study OR evaluation studies OR follow-up studies OR prospective studies OR cross-over studies OR control* OR prospectiv* (OR volunteer* NOT animal) AND (third molar) AND (antibiotic OR amoxicillin OR clavula*) AND (infection OR dry socket) AND (extraction OR removal). For the Spanish language databases, we used the following Spanish terms: (antibiótico OR amoxicilina OR clavulan*) AND (infección OR alveolitis seca) AND (exodoncia OR extracción). The references in each paper were reviewed, and we also searched for conference abstracts.

- Selection of studies: Two researchers independently performed the searches in the databases with the aforementioned criteria. After applying the filters, we obtained the following: 26 papers from PubMed; 123 from SCOPUS; 668 from ScienceDirect; 69 from the Web of Science; 65 from Evidence-Based Dentistry, 42 from the Cochrane Library, 4 from TESEO, and 2 from IME-Biomedicina (a CSIC database). The databases not listed did not yield any relevant publications. Having removed duplicate publications and any for which it was clear from the title and abstract that they did not met the inclusion criteria, 75 papers were retrieved, and these were reviewed by two of the authors.  Table 1 summarises the 65 studies excluded and the reasons for their exclusion. Qualitative and quantitative analysis was conducted considering the remaining 10 papers (RDBPC studies).

**Table 1:**
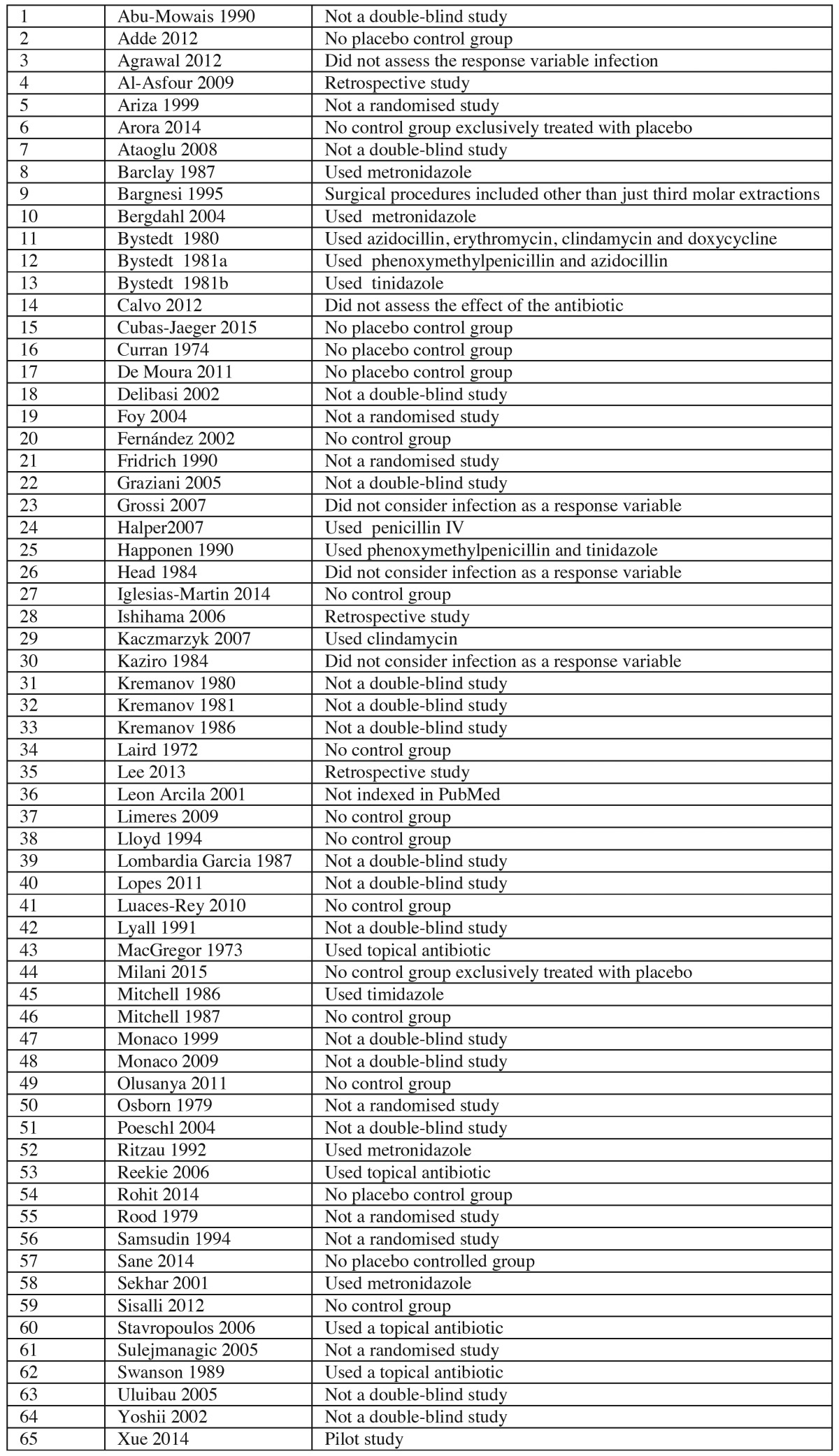
Studies excluded from the meta-analysis and the reasons for their exclusion.

- Data extraction process: Data were extracted on 13 variables from each of the studies (10-19). Each study was examined independently by two researchers.

- List of data: The types of data collected are listed in  Table 2 and  Table 2continue.

**Table 2:**
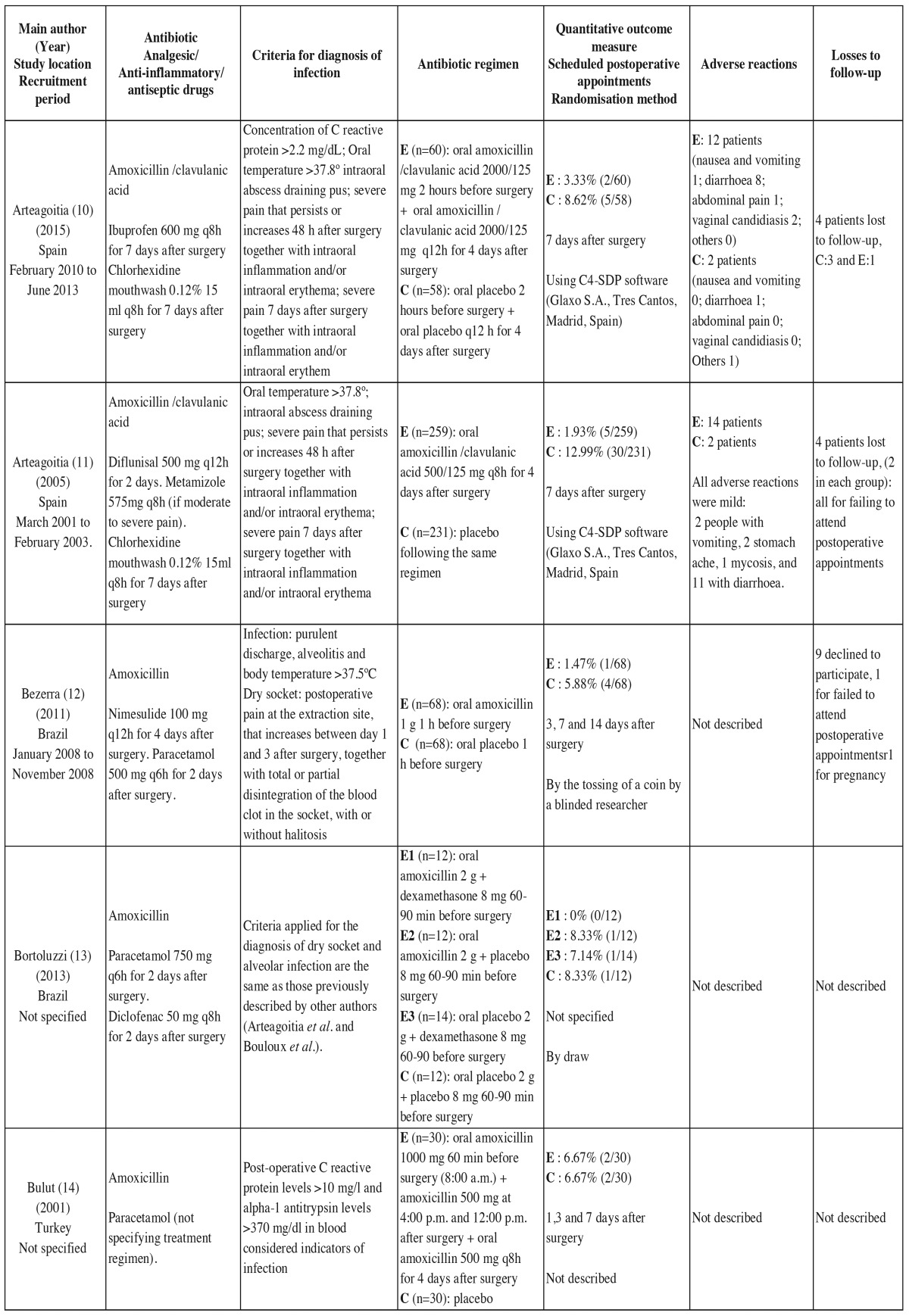
Main characteristics of the 10 randomized double-blind placebo-controlled studies included in the qualitative analysis.

Risk of bias in individual studies. Qualitative and quantitative data were collected on potential sources of bias in each of the studies ( Table 2 and  Table 2continue ). To assess bias in each study, we considered the following factors:

**Table 2continue:**
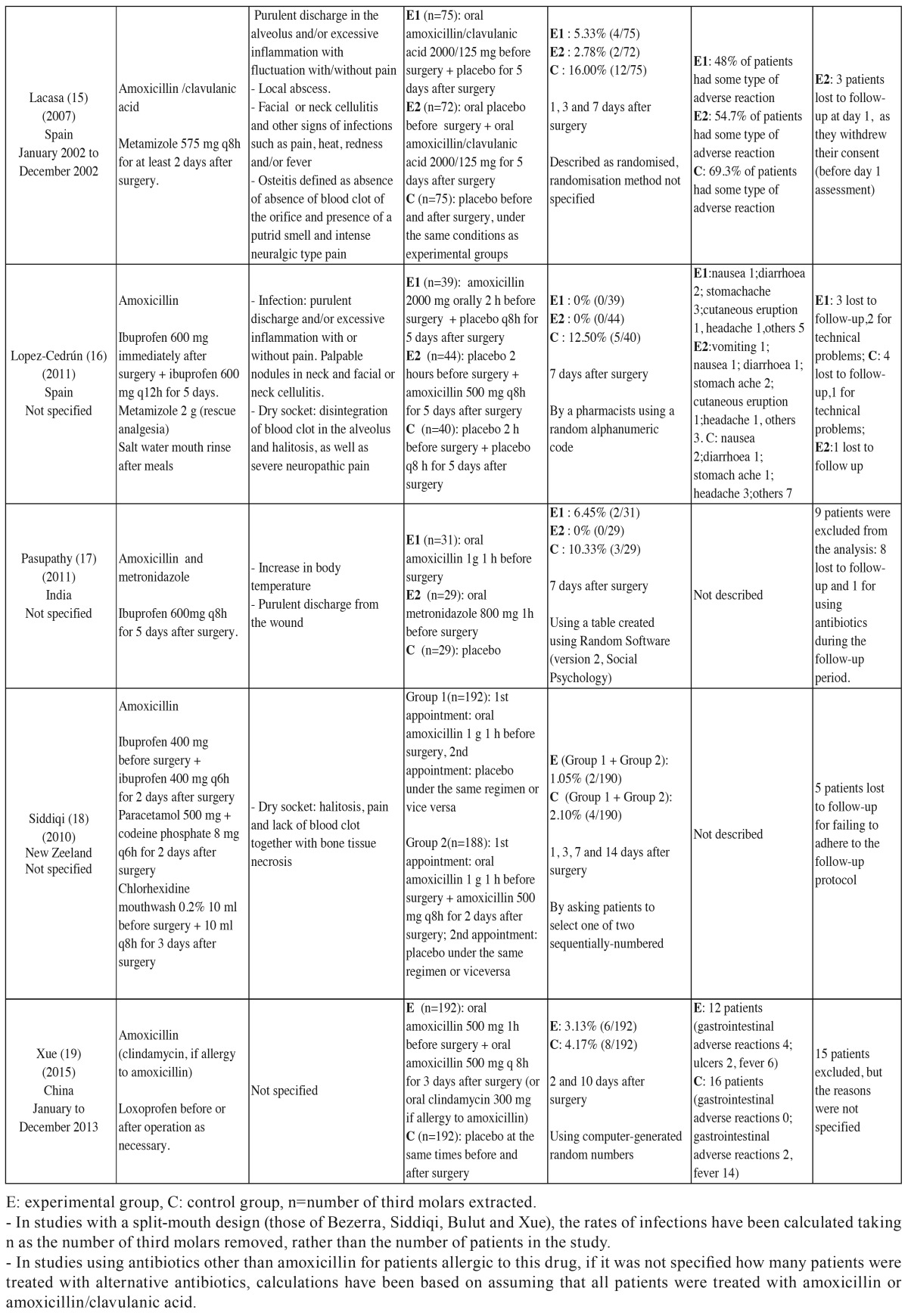
Main characteristics of the 10 randomized double-blind placebo-controlled studies included in the qualitative analysis.

1- Generation of the random sequence: we assessed whether the method for generating the random sequence was appropriate.

2- Concealment of allocation: we assessed how patients were allocated to each group (experimental and control) and how allocation was concealed.

3- Blinding of participants and personnel: all the studies included claimed to be double blind, but they did not report the method for blinding of participants and personnel.

4- Blinding of outcome assessment: as above, though all the studies included claimed to be double blind, they did not specify the method for blinding assessment of the outcome.

5- Handling of data: we identified whether patients lost to follow-up were included in the analysis and whether the analysis was carried out on an intention-to-treat basis.

6- Selective reporting: we checked whether data were in fact reported for all the variables and outcomes that authors had planned to report a priori.

7- Other sources of bias: we sought to identify other potential sources of bias.

For each study, all of these factors were analysed and the study was then assigned to one of three categories (low risk, unclear risk, high risk) based on the estimated risk of bias.

- Summary measure of efficacy: the analysis of efficacy was based on the relative risk (RR) or cumulative incidence ratio in the treatment vs. control groups. In clinical trials that compared several experimental groups using the same antibiotic under different regimens with a single control group, the data considered were the total numbers of surgical interventions and complications in the experimental groups, without considering them as independent clinical trials. To assess the clinical significance of the treatment effect, we used the difference in the incidence or attributable risk of infection and calculated the number needed to treat (NNT) to prevent one case of infection.

- Synthesis of results: All the analyses were carried out using StataCorp 2013 Stata Statistical Software: Release 12 (College Station, TX: StataCorp LP) and Review Manager (RevMan) Version 5.2 (Copenhagen: The Nordic Cochrane Centre. The Cochrane Collaboration, 2012). We studied the heterogeneity of the different studies using the I2 statistic, an expression related to Cochran’s Q test. The overall relative risk, the result from combining data from the different studies, was calculated using an inverse-variance approach with a random effect model. Empirical correction was used for the studies with zero effect sizes in one of their arms. Any studies with a zero effect size in both arms were excluded. The clinical significance was analysed by calculating the NNT for each study and overall.

- Risk of between-study bias. The publication bias was assessed graphically using a funnel plot and quantitatively with the methods of Egger and Macaskill. The number of unpublished studies was estimated with Rosenthal’s method.

- Additional analysis: We also carried out meta-analysis stratified by the type of antibiotic and cumulative meta-analysis by publication date, as well as analysing adverse reactions.

## Results

**Figure 1:**
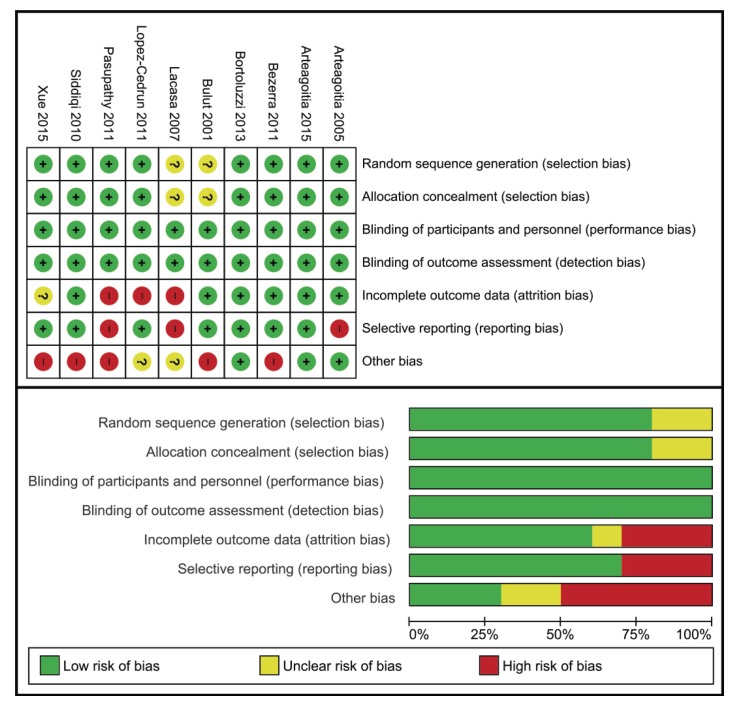
1. Risk of bias in studies included in the systematic review. - All the studies were considered low risk in terms of performance and detection bias, given that a double-blind design was a selection criterion for the meta-analysis. - In studies with a split-mouth design, it was considered that that there might be other sources of bias given the duration of the washout period (no more than 4 weeks in all cases).

**Figure 2:**
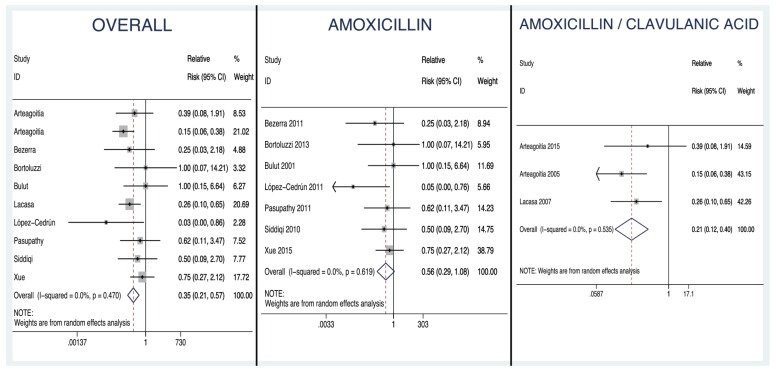
Forest Plots. Overall forest plot: graphical representation of the estimates of the RRs and 95% CIs based on the samples in each of the studies, including both those that used amoxicillin and those that used amoxicillin/clavulanic acid, together with their relative weights. Amoxicillin forest plot. Amoxicillin/clavulanic acid forest plot.

- Selection of studies: Out of the 75 studies retrieved, qualitative and quantitative analysis was performed on 10 RDBPC studies.  Table 1 lists the studies excluded and the reasons for their exclusion.

- Characteristics of the studies:  Table 2 and  Table 2continue lists the main characteristics of the 10 RDBPC studies published between 2001 and June 2015 that were included in the qualitative and quantitative analyses.

- Risk of bias in the studies: (Fig. 1) illustrates the estimated risk of bias in each of the studies. Despite potential sources of bias having been identified, none of the RDBPC studies were excluded for this reason.

- Results of the individual studies: The forest plot (Fig. 2) is a graphical representation of the estimates of the RRs and 95% CIs based on the samples in each of the studies, together with their relative weights. Forest plots are shown for the overall analysis and for the analysis stratified by antibiotic (amoxicillin or amoxicillin/clavulanic acid).

- Synthesis of the results:

Analysis of the overall efficacy of amoxicillin with or without clavulanic acid: The quantitative analysis included 1997 extractions: 1072 in experimental groups and 925 in control (placebo) groups, with 27 and 74 reported events of dry socket and/or infection respectively. The overall RR was found to be 0.350, with a 95% CI of 0.214 to 0.574, this being significant (*p*<0.0001) and different from 1, indicating that treatment with amoxicillin with or without clavulanic acid prevents the development of infectious complications (dry socket, infection, or both conditions concurrently).

**Figure 3:**
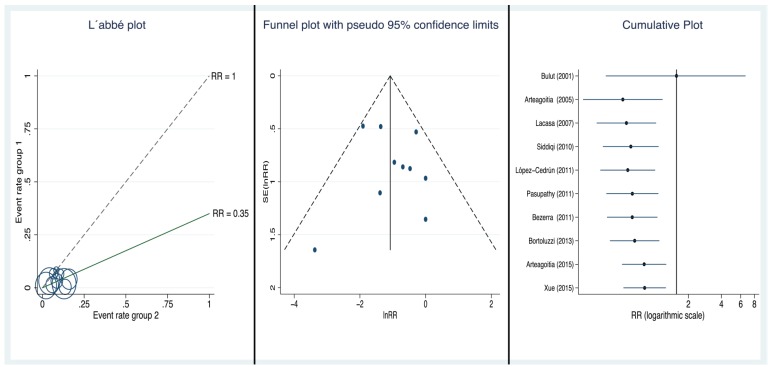
L'Abbé plot, Funnel plot, Cumulative plot.

Analysis of the heterogeneity: The Q statistic was 8.65 and I2 was 0% (*p*=0.470), supporting the assumption of homogeneity among the studies. Further, there is no sign of heterogeneity in the L’Abbé plot (Fig. 3), all the circles being grouped close together, independently of their size and baseline risk.

Analysis of clinical significance: The NNT for each of the studies is reported in  Table 3 and the overall NNT, adjusting for the weight of each study, was estimated to be 18 (95% CI 13 to 29). This means that we would need to treat between 13 and 29 patients with amoxicillin with or without clavulanic acid to prevent one case of infection.

**Table 3:**
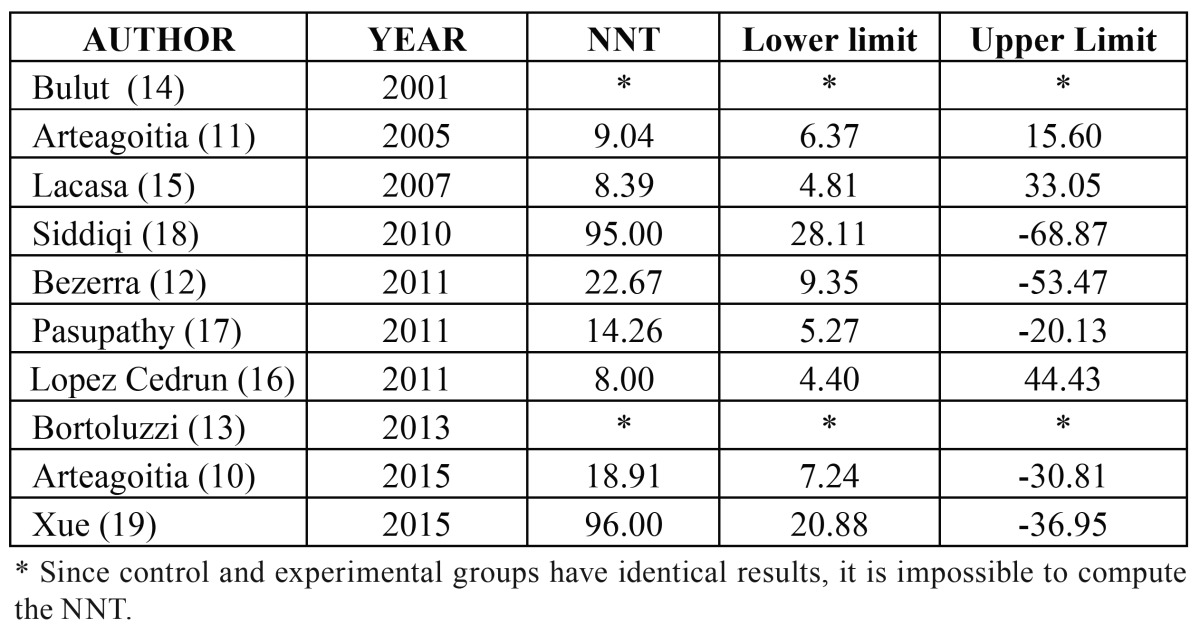
Number needed to treat (NNT) for each individual study included in the meta-analysis.

- Risk of publication bias: The funnel plot (Fig. 3) is not absolutely symmetrical around the summary estimate RRw, and hence, given the suspicion that there may be publication bias, we carried out the corresponding quantitative analysis.

The Begg method suggested a lack of publication bias (Kendall´s Tau being 0.1556; *p*=0.59), and Egger´s more sensitive method also suggested a lack of publication bias (with an intercept value of 0.4772, which is not significant, *p*=0.627). Macaskill’s more specific procedure yielded a slope that was close to 0 and non-significant (*p*=0.489), confirming the lack of publication bias, both when using the sample size (n) as the independent variable, as proposed by Peters, and when the regression uses the inverse of the sample size (1/n) as the independent variable (*p*=0.330). Lastly, with Rosenthal’s method, it was estimated that it would be necessary to add 79 non-significant studies to cause the results of this meta-analysis to become non-significant.

- Additional analysis:

Stratified analysis: We analysed independently the studies in which the treatment was amoxicillin or amoxicillin/clavulanic acid:

- Amoxicillin: We included 7 studies (1167 extractions: 606 in experimental groups and 561 in controls with 14 and 27 events of dry socket and/or infection respectively). The RR was 0.563 (*p*=0.082, 95% CI 0.295 to 1.08). We found no evidence of heterogeneity (I2=0.00%, *p*=0.619). The NNT was 40, meaning that about one in every 40 patients would benefit from the treatment. The 95% confidence interval for the NNT ranged from 22 to 274.

- Amoxicillin/clavulanic acid: We included 3 studies (830 extractions: 466 in experimental groups and 364 in controls with 13 and 47 events of dry socket and/or infection respectively). The RR was 0.215 (*p*<0.001, 95% CI 0.117 to 0.395). Again, we found no evidence of heterogeneity (I2=0.00%, *p*=0.535). The NNT was 10, meaning that about one in every 10 patients would benefit from the treatment, and the 95% confidence interval for the NNT ranged from 7 to 16.

Cumulative analysis: Figure 3 shows the evolution of the 95% CI of the weighted estimate in the cumulative meta-analysis by year of publication, that is, as we added RDBPC studies to the analysis in date order. It can be observed that the first study found a non-significant association but that with the progressive addition of the studies conducted to date the RR increased towards 1.

Analysis of adverse reactions: Five studies reported adverse reactions ( Table 2 and  Table 2 continue) with a total follow-up of 1337 patients (741 in experimental groups and 596 controls). A total of 222 patients had some type of adverse reaction associated with the antibiotic given (136 in experimental groups and 86 controls). The RR was 1.188 (95% CI 0.658 to 2.146; *p*=0.567). The adverse reactions were generally mild and short lived. The number needed to harm (NNH) was 26, meaning that 1 in 26 patients given the prophylactic antibiotics would have an adverse reaction.

## Discussion

Our meta-analysis includes 10 RDBPC clinical trials that assess the efficacy of amoxicillin with and without clavulanic acid to prevent dry socket, infection and both conditions concurrently after third molar extraction. These studies yielded a total of 1997 third molar extractions. We only selected trials that used placebo in the control group. It is important to highlight that we have not taken into account the antibiotic regimen used. As noted in the qualitative analysis, the studies included are not free from individual bias, but we have not detected publication bias. Adverse reactions were more frequent in the experimental group but were generally mild.

In the quantitative analysis, we used a multiplicative relative risk model and estimates were weighted by the inverse of the variance. We opted to use a random effect model, which assigns a fixed constant coefficient of variability to all studies, and this gives more importance to studies with smaller sample size; however, this is the most appropriate type of model when analysing fewer than 20 studies, provided there is no publication bias.

We have carried out analysis stratifying by the type of antibiotic (amoxicillin or amoxicillin with clavulanic acid), finding this variable to be relevant. The hypothesis of the analysis by subgroups was established a priori and the variable type of antibiotic used for weighing was defined prior to randomisation in all of the studies. This stratified meta-analysis has found that the statistical significance of the RR differs between the subgroups: in the case of amoxicillin alone, the CI of the RR (95% CI 0.295 to 1.08) includes 1 and the result was not significant (*p*=0.082), while for amoxicillin and clavulanic acid, the result was statistically significant (*p*<0.001, 95% CI 0.117 to 0.395). We should emphasize that only three studies were included in the amoxicillin/clavulanic acid subgroup analysis, and that two of these were conducted by the same research team (10,11), which could be a source of bias.

We have not used restrictive criteria in the definition of infection or dry socket. We found significant differences in the rate of infection and/or dry socket in the groups treated with placebo. This may be attributable to differences in the diagnostic criteria, or factors related to the technique used, surgeon experience, asepsis or patient characteristics. In our meta-analysis, the mean rate of infection in the control group was 8%, with very different results across the clinical trials included. The mean rates of infection in the placebo group were 5% in studies using amoxicillin and 13% in studies using amoxicillin/clavulanic acid.

The rate of infection was not significantly different in patients given amoxicillin (2.31%) or amoxicillin/clavulanic acid (2.79%). In contrast, there were notable differences analysing absolute risk reduction, with values of 2.50% (95% CI 0.37 to 4.64%) in the amoxicillin group and 10.12% (95% CI 6.37 to 13.88%) in the amoxicillin/clavulanic acid group. This discrepancy is understandable given the difference in rates of the conditions considered in patients treated with placebo in the two subgroups, with higher rates of infection in the case of studies using amoxicillin/clavulanic acid than those using amoxicillin ( Table 2 and  Table 2continue ). This underlines the fact that failing to include placebo groups in trials of antibiotics may lead to different conclusions regarding drug efficacy (4).

Other meta-analyses have been published on the efficacy of antibiotics for the prevention of inflammatory and infectious complications after third molar extraction. In 2007, Ren *et al.* (9) studied the efficacy of antibiotic prophylaxis including 15 clinical trials. They did not limit the search to double-blind studies, included different families of antibiotics and analysed the efficacy taking into account the treatment regimen, concluding that the antibiotic treatment is effective only when used before surgery. In 2012, Lodi *et al.* (6) included 18 double-blind randomised clinical trials and analysed different families of antibiotics and several different response outcomes (infection, dry socket, pain, inflammation, trismus and high temperature). They concluded that, compared to placebo, antibiotics (without specifying which) reduce the risk of infection by 70%, a very similar result to ours (65%), and that of dry socket by 38%. On the other hand, they found that antibiotics are associated with an increase in adverse effects compared to placebo (RR 1.98; 95% CI 1.10 to 3.59; *p* = 0.02). In our case, the relative risk was somewhat lower, but only half of the studies had recorded adverse reactions (RR = 1.188; 95% CI 0.658 to 2.146; *p* =0.567). Lodi *et al.* (6) estimated that, despite the results obtained, physicians should consider whether treating 12 patients with antibiotics to prevent one case of infection does more harm than good.

In our case, the results should also make us think. To avoid one patient having an infectious complication using amoxicillin prophylactically, we would need to treat 40 patients. Moreover, as mentioned earlier, amoxicillin does not significantly reduce the risk of infection and/or dry socket. For this reason, we believe that its use is not justified.

In the case of amoxicillin/clavulanic acid, we would have to treat 10 patients to avoid 1 case of infectious complication. It is important to analyse the clinical significance of these results. First, we should take into account the low rate of infectious complications and the lack of serious complications. On the other hand, the risks of antibiotic use are widely documented, in relation to increases in antibiotic resistance at the population level (20,21), as well as adverse reactions at the individual level. In this meta-analysis, 1 out of 26 patients treated with amoxicillin with or without clavulanic acid had some type of adverse reaction.

For all these reasons, and given our results, we conclude that there no basis for recommending the prophylactic use of amoxicillin without clavulanic acid for preventing infection and/or dry socket after third molar extraction in healthy patients. Regarding amoxicillin/clavulanic acid, although the null hypothesis was rejected and prophylactic use was statistically significantly effective, taking into account the NNT, low rate of infectious complications, adverse reactions in experimental groups and lack of serious complications reported in controls, the prescription of this combination of antibiotics cannot be justified either.
